# Comprehensive Molecular Characterizations of Chinese Patients With Different Subtypes of Lung Squamous Cell Carcinoma

**DOI:** 10.3389/fonc.2020.607130

**Published:** 2020-12-10

**Authors:** Jie Qian, Rongrong Chen, Ruiying Zhao, Yuchen Han, Yongfeng Yu

**Affiliations:** ^1^ Shanghai Lung Cancer Center, Shanghai Chest Hospital, Shanghai Jiao Tong University, Shanghai, China; ^2^ Department of Internal medicine, Huadong Hospital, Fudan University, Shanghai, China; ^3^ Department of Pathology, Shanghai Chest Hospital, Shanghai Jiao Tong University, Shanghai, China

**Keywords:** lung squamous cell carcinoma, histopathological subtypes, genetic profiles, prognosis factors, next-generation sequencing

## Abstract

**Background:**

This study aims to profile integrative genomic spectra of Chinese patients with different subtypes of lung squamous cell carcinoma (LUSC) and explore potential molecular prognosis factors.

**Methods:**

We retrospectively identified 204 surgically resected LUSC patients in Shanghai Chest Hospital who underwent capture-based targeted next-generation sequencing (NGS) with a panel of 68 lung cancer‐related genes from September 2017 to January 2019. NGS was used to profile comprehensive molecular characterizations.

**Results:**

Of 204 cases, 114 (55.9%) were keratinizing squamous cell carcinoma (KSCC), 77 (37.7%) were non-keratinizing squamous cell carcinoma (NKSCC), 13 (6.4%) were basaloid squamous cell carcinoma (BSCC), respectively. All subtypes presented similarly high proportions of mutations, including TP53, CDKN2A, and NOTCH1. A comparable prevalence of FGFR1 amplifications was identified between KSCC and NKSCC (11.4 *versus* 26.9%, p = 0.007). Compared with NKSCC, IGF1R amplifications were more frequent in BSCC (0 *versus* 15.4%, p = 0.019). We found cases with TP53 alterations had less EGFR alterations in KSCC (P = 0.013, OR = 0.158). Compared with TCGA cohorts, our Chinese cohorts exhibited statistic differences in both somatic mutations and signaling pathways. We found that STK 11 alterations and TOP2A alterations were significantly associated with higher risk of recurrence in patients with LUSC.

**Conclusions:**

Significant differences exist among three subtypes of LUSC in molecular characterizations.

## Introduction

Lung cancer has been the malignant tumor with highest incidence and mortality worldwide (1, 2). As the most prevalent type of lung cancer, non‐small cell lung cancer (NSCLC) comprises two main histological types: lung adenocarcinoma (LUAD) and lung squamous cell carcinoma (LUSC). Second to LUAD, LUSC accounts for approximately 25–30% of lung cancer (3). With the emergence and progress of molecular targeted therapies, the objective response rates (ORRs) and progression-free survival (PFS) of NSCLC patients treated with gene-directed therapies have been improved compared with traditional cytotoxic chemotherapy (4).

Targeted therapies have mainly been applied to patients with LUAD, most of which are never-smokers and women. Contrarily, as a type proved to closely associate with chronic tobacco exposure, druggable driver mutations are rare and therapeutic effects of targeted therapies are limited in LUSC. As a result of these, routine genetic testing is even not recommended for LUSC in clinical practice (5). Contrast to numerous studies on molecular characteristics of LUAD, little is known about genetic profiles of LUSC, which might be responsible for sluggish progress of targeted therapies in LUSC.

According to the 2015 World Health Organization (WHO) Classification of Tumors of the Lung, LUSC was reclassified as keratinizing squamous cell carcinoma (KSCC), non-keratinizing squamous cell carcinoma (NKSCC), and basaloid squamous cell carcinoma (BSCC) (6). Previous researches had outlined a comprehensive genomic profiling of LUSC in Caucasian and East Asian patients (7–9), whereas, there are few studies on molecular characteristics of LUSC in Chinese patients based on the 2015 WHO Classification to date. Hence, in this study, we retrospectively compared and analyzed the clinicopathologic, genetic characteristics, and prognosis of 204 LUSC patients who had received next-generation sequencing (NGS) testing in the same platform during the same period, aiming to reveal the potential associations of genetic profiles and LUSC subtypes. It may provide theoretical evidences for precision medicine of LUSC.

## Material and Methods

### Patients and Samples

Between September 2017 and January 2019, we retrospectively reviewed the clinic data of 204 surgically resected LUSC patients who had received NGS assay in Shanghai Chest Hospital. According to the 2015 WHO classification, all formalin‐fixed, paraffin‐embedded hematoxylin and eosin-stained tumor tissues were verified by two independent pathologists to confirm the diagnosis of LUSC subtypes. Pathologic staging was assessed based on the eighth edition of the tumor, node and metastasis (TNM) classification for lung cancer. This study has been approved by the institutional review board of Shanghai Chest Hospital. Tissue DNA Extraction

Formalin-fixed, paraffin-embedded tumor samples were reviewed by qualified pathologists to ensure that those tissues containing sufficient (at least 10%) tumor cells by H&E staining were qualified for DNA extraction. DNA was isolated and extracted from the tumor tissues using QIAamp DNA FFPE tissue kit (Qiagen, Hilden, Germany) according to the manufacturer’s instructions. A minimum of 50 ng of DNA is eligible for NGS library construction.

### Capture-Based Targeted DNA Sequencing

DNA was sheared using Covaris M220 (Covaris, MA, USA), followed by end repair, phosphorylation and adaptor ligation. Fragments of size 200–400 base pairs (bp) were selected by bead (Agencourt AMPure XP Kit, Beckman Coulter, Brea, CA, USA), followed by hybridization with capture probes baits, hybrid selection with magnetic beads, and polymerase chain reaction (PCR) amplifications. DNA concentration and genomic DNA quality were measured by Qubit 2.0 fluorometer with the dsDNA high-sensitivity assay kit (Life Technologies, Carlsbad, CA, USA) and 260 nm/280 nm absorption ratio, respectively. Indexed samples were sequenced on Nextseq500 sequencer (Illumina, Inc, Madison, WI, USA) with pair-end reads. The genomic proﬁles were assessed using Lung Core panel from Burning Rock Biotech (Guangzhou, China), which consists of the whole exons of 68 lung cancer-related genes (the list of genes was provided in **Supplemental Table 1**) and spans 345 kb of the human genome.

### Sequence Data Analysis

Sequence data were mapped to the reference human genome (hg19) using Burrows‐Wheeler aligner v.0.7.10 (10). Local alignment optimization, variant calling, and annotation were performed using Genome Analysis Tool Kit v.3.2 (11) and VarScan (12). Variants were filtered using the VarScan. Loci with depth less than 100 were filtered out. Minimal of five supporting reads were needed for INDELs and eight supporting reads were needed for SNV calling. According to the ExAC, 1000 Genomes, dbSNP, ESP6500SI-V2 database, variants with population frequency over 0.1% were grouped as SNP and excluded from further analysis. Remaining variants were annotated with ANNOVAR (13) and SnpEff v3.6 (14). DNA translocation analysis was performed using both Tophat2 and Factera 1.4.3 (15).

### Statistical Analysis

Statistical analysis was carried out with the SPSS (version 24.0, SPSS Inc, Chicago, IL, USA) and Prism software (version 7.0, GraphPad Software, San Diego, CA, USA). Pearson’s chi‐squared test and Fisher’s exact test were used to assess differences of mutation frequencies among three subtypes and other clinical characteristics. All bioinformatics analyses were performed with R software (version 3.4.0, the R Foundation for Statistical Computing, Vienna, Austria). P value less than 0.05 and OR greater than 2 or less than 0.5 were listed. Non-negative matrix factorization algorithm was used to cluster genomic profile by R package NMF. Disease-free survival (DFS) was defined as the time from surgery to recurrence. DFS curves were analyzed using the Kaplan–Meier method. The identification of prognostic factors for DFS was carried out using bivariate Cox proportional hazards models. Parameters with a p-value less than 0.1 in univariate analysis were evaluated in a Cox proportional hazards multivariable model. Results were expressed as hazard ratio (HR) with 95% confidence intervals (CI). Two-tailed P value <0.05 was considered to be statistically significant.

## Results

### Baseline Clinical Characteristics

A total of 204 patients included 114 (55.9%) cases with KSCC, 77 (37.7%) cases with NKSCC, and 13 (6.4%) cases with BSCC. The overwhelming majority of the patients were males (92.2%), former or current smokers (80.4%), and older people (75.5%). In the cohort, 35.8% (73/204) were diagnosed with stage I, 36.3% (74/204) with stage II, and 27.9% (57/204) with stage III/IV. The detailed clinical features of the patients were listed in **Table 1**. Apparently, significant differences existed in gender (p = 0.004) and smoking history (p = 0.009) among the LUSC subtypes. Further analysis showed that compared with NKSCC, there were more male (p = 0.002) and former or current smokers (p = 0.005) in KSCC, whereas there was no significant correlation between non-BSCC and BSCC. The statistical analysis did not show any significant associations between other clinical characteristics and pathologic subtypes in our study.

**Table 1:** Clinical characteristics of 204 LUSC patients with NGS assay.

Characteristics	Total	KSCC	NKSCC	BSCC	P value
	(n = 204)	(n = 114)	(n = 77)	(n = 13)	
**Gender**					
Male	188 (92.2%)	111 (97.4%)	65 (84.4%)	12 (92.3%)	0.004
Female	16 (7.8%)	3 (2.6%)	12 (15.6%)	1 (7.7%)	
**Age**					
≥60	154 (75.5%)	89 (78.1%)	55 (71.4%)	10 (76.9%)	0.583
<60	50 (24.5%)	25 (21.9%)	22 (28.6%)	3 (23.1%)	
**Smoking status**					
Non-smoker	40 (19.6%)	14 (12.3%)	23 (29.9%)	3 (23.1%)	0.009
Smoker	164 (80.4%)	100 (87.7%)	54 (70.1%)	10 (76.9%)	
**Stage**					
I	73 (35.8%)	47 (41.2%)	23 (29.9%)	3 (23.1%)	0.200
II	74 (36.3%)	37 (32.5%)	29 (37.7%)	8 (61.5%)	
III/IV	57 (27.9%)	30 (26.3%)	25 (32.5%)	2 (15.4%)	

Gender: KSCC versus NKSCC (p = 0.002), KSCC versus BSCC (p = 0.35),

Smoking history: KSCC versus NKSCC (p = 0.005), KSCC versus BSCC (p = 0.38), NKSCC versus BSCC (p = 0.75).

LUSC, lung squamous cell carcinoma; NGS, next‐generation sequencing; KSCC, keratinizing squamous cell carcinoma; NKSCC, non-keratinizing squamous cell carcinoma; BSCC, basaloid squamous cell carcinoma.

### Characterization of Genetic Alterations

**Figure 1:**
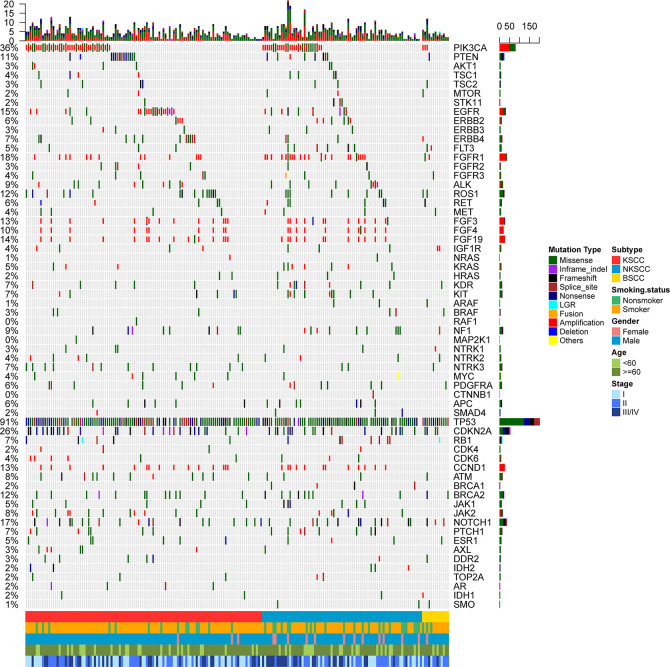
Mutational spectrum of the LUSC patients grouped according to histological subtypes. Smoking status, gender, age, and disease stage were also annotated at the bottom of the oncoprint. Each column represents a patient and each row represents a gene. Column on the left represents the mutation rate of each gene. Column on the right represents mutated genes. Top plot represents the overall number of mutations a patient carried. Different colors denote different types of mutation. KSCC, keratinizing squamous cell carcinoma; NKSCC, non-keratinizing squamous cell carcinoma; BSCC, basaloid squamous cell carcinoma.

Totally, we analyzed 204 surgically resected LUSC tissue samples tested with NGS panel of 68 cancer-related genes. The comprehensive genetic spectra of patients with LUSC showed that the overwhelming majority of them (99.5%) harbored genetic abnormalities including somatic mutations or copy number alterations (CNAs). 91% of patients harbored TP53 alterations, among which all BSCC patients harbored TP53 alterations. Second to TP53, 36% of patients harbored PIK3CA alterations and 26% harbored CDKN2A alterations (**Figure 1**).

We also explored the differences in genetic alterations based on clinical characteristics, including stage, gender, smoking status, and age. FGFR alterations were more common in patients diagnosed with stage II, compared with stage I and stage III/IV (5.4% in stage II, 0% in stage I, 0% in III/IV, p = 0.038). In female cases, mutation frequencies of SMAD4, FLT3, and ARAF were higher (p = 0.017, p = 0.034, p = 0.017, respectively), while TP53 were lower (p = 0.005). Contrast to non-smoker, TP53 mutations were more frequent in smoker (77.5 *vs* 95.1%, p = 0.001). AXL mutations occurred more frequently in younger patients than older patients (6.0 *vs* 0.6%, p = 0.046). Correlations between genetic alterations and clinical characteristics are summarized in **Supplemental Table 6**–**9**.

### Characterization of Somatic Mutations

**Figure 2:**
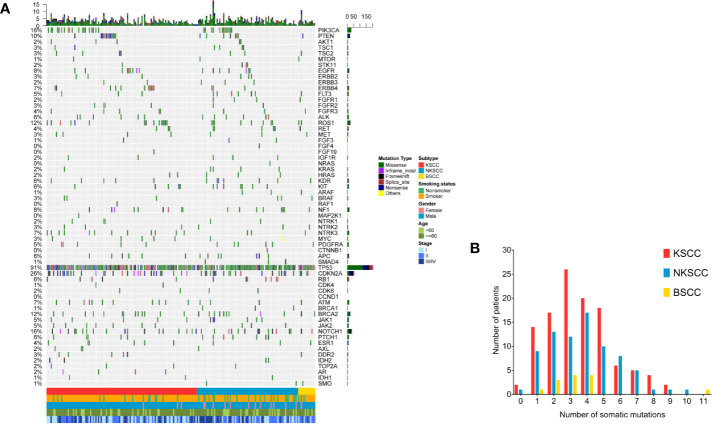
**(A)** The spectra of somatic mutations of the LUSC patients grouped according to histological subtypes. Smoking status, gender, age, and disease stage were also annotated at the bottom of the oncoprint. Each column represents a patient and each row represents a gene. Column on the left represents the mutation rate of each gene. Column on the right represents mutated genes. Top plot represents the overall number of mutations a patient carried. Different colors denote different types of mutation **(B)**. Somatic mutation frequency of LUSC patients according to their histological subtypes. X-axis denotes number of somatic mutations, Y-axis denotes number of patients. LUSC, lung squamous cell carcinoma; KSCC, keratinizing squamous cell carcinoma; NKSCC, non-keratinizing squamous cell carcinoma; BSCC, basaloid squamous cell carcinoma.

In summary, at least one somatic mutation existed in 201 (98.5%) patients, among which 112 (98.2%) were KSCC, 76 (98.7%) were NKSCC, 13 (100%) were BSCC respectively. We could see that most patients harbored multiple somatic mutations either in a monogene or different genes (**Figures 2A, B**). The TOP 20 common somatic mutations detected in LUSC, KSCC, NKSCC, and BSCC were demonstrated in **Supplemental Figures 1A–D**, among which the mutation frequency of CDKN2A loss was 17.6% in LUSC, 16.7% in KSCC, 18.2% in NKSCC, and 23.1% in BSCC; JAK2 loss was 4.4% in LUSC, 6.1% in KSCC, 2.6% in NKSCC, and 0 in BSCC. Of note, cases harboring JAK2 loss were associated with concurrent CDKN2A losses in LUSC (33.3%, 3 of 9), KSCC (28.6%, 2 of 7), and NKSCC (50%, 1 of 2). All the subtypes presented similarly high proportions of mutations, including TP53 (93.9% in KSCC, 88.5% in NKSCC, 100% in BSCC, p = 0.25), CDKN2A (28.1% in KSCC, 25.6% in NKSCC, 30.8% in BSCC, p = 0.88), and NOTCH1 (15.8% in KSCC, 15.4% in NKSCC, 30.8% in BSCC, p = 0.40). However, compared with BSCC (0%), PIK3CA mutations occurred more frequently in KSCC (16.7%) and NKSCC (16.7%) (p = 0.36).

As shown in **Supplemental Tables 2**–**5**, statistic differences among LUSC pathologic subtypes were enriched in somatic mutations including FGFR1 (p = 0.035), SMO (p = 0.038), and ARAF (p = 0.038). However, Detailed analysis showed no statistic differences between any two subtypes.

### Characterization of Copy Number Alterations (CNAs)

**Figure 3:**
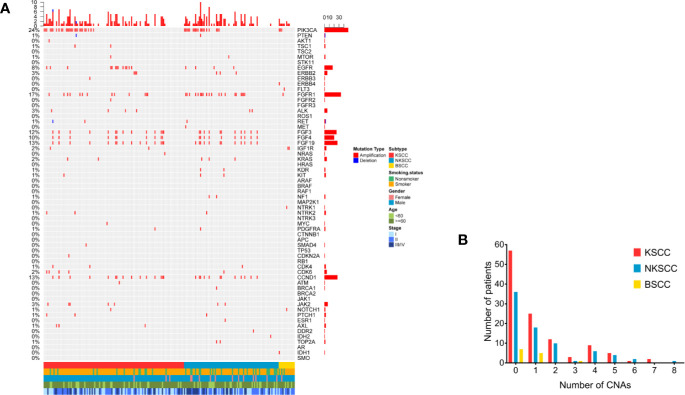
**(A)** The spectra of copy number alterations of the LUSC patients grouped according to histological subtypes. Smoking status, gender, age, and disease stage were also annotated at the bottom of the oncoprint. Each column represents a patient and each row represents a gene. Column on the left represents the alteration rate of each gene. Column on the right represents genes. Top plot represents the overall number of alterations a patient carried. Different colors denote different types of alteration **(B)**. Copy number alterations frequency of LUSC patients according to their histological subtypes. X-axis denotes number of copy number alterations, Y-axis denotes number of patients. LUSC, lung squamous cell carcinoma; KSCC, keratinizing squamous cell carcinoma; NKSCC, non-keratinizing squamous cell carcinoma; BSCC, basaloid squamous cell carcinoma.

Of the 204 LUSC patients identified, as shown in **Figures 3A, B**, 103 (50.5%) harbored CNAs, among which KSCC were 57 (50.0%), NKSCC were 40 (51.9%), KSCC were 6 (46.2%), respectively. PIK3CA amplifications were most prevalent in LUSC (24%), followed by FGFR1 amplifications (17%). To sum up, the most commonly detected CNAs in LUSC, KSCC, NKSCC, and BSCC were depicted in **Supplemental Figures 2A–D**.

As shown in **Supplemental Tables 2**–**5**, the differences of CNAs in LUSC pathologic subtypes were statistically significant as followed: FGFR1 amplifications (p = 0.015), IGF1R amplifications (p = 0.011). A comparable prevalence of FGFR1 amplifications was identified between KSCC and NKSCC (11.4 *vs* 26.9%, p = 0.007). Compared with NKSCC, IGF1R amplifications were more frequent in BSCC (0 *vs* 15.4%, p = 0.019). Notably, amplifications of genes at 11q13 involving CCND1 (10.5% in KSCC, 14.1% in NKSCC, 0% in KSCC), FGF3 (11.4% in KSCC, 12.8% in NKSCC, 0% in KSCC), FGF4 (8.8% in KSCC, 11.5% in NKSCC, 0% in KSCC) and FGF19 (10.5% in KSCC, 10.3% in NKSCC, 0% in KSCC) displayed differences between BSCC and non-BSCC, however, the results were not statistically different.

### Characterization of Signaling Pathway

Compared with KSCC, incidence of RAS signaling pathway mutations in NKSCC was more frequent (14.9 *vs* 26.0% p = 0.087) (**Supplemental Figure 3A**). As for other signaling pathways including PIK-AKT-mTOR pathway, TP53-cell cycle, Receptor tyrosine kinase pathway, MAPK signaling pathway, Wnt signaling pathway, and Homologous recombination, there showed no differences among three subtypes. Details of signaling pathway maps were shown in **Supplementary Figures 3B–H**.

### Exclusivity and Co-occurrence of Mutations; NMF Cluster Analysis

**Figure 4:**
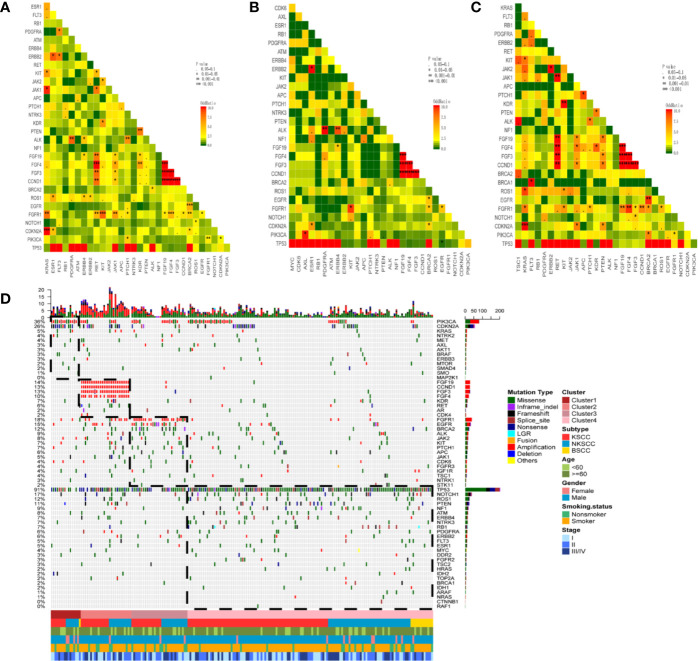
Pairwise assessment of mutual exclusivity and association in driver genes of LUSC **(A)**, KSCC **(B)**, NKSCC **(C)**, respectively. The red color is associated with a positive correlation whereas green indicates mutual exclusivity. Asterisks indicate significant relationships. Co-occurrence and exclusivity of the genes in our cohort (n = 204 patients) were assessed using the Fisher’s exact test **(D)**. NMF clustering of all genes based on the somatic mutations and CNAs from 204 cases. Hierarchical clustering revealed the cluster 1 (n=16) composed by the majority of PIK3CA and CDKN2A mutations, the cluster 2 (n = 27) grouped samples with CCND1/FGF19/FGF3/FGF4 amplifications, the cluster 3 (n = 30) consisted samples with FGFR1 amplifications, EGFR, BRCA2 mutations and the cluster 4 (n = 131) was constituted by samples with BSCC subtype, TP53, NOTCH1, ROS1, PTEN, NF1 mutations. LUSC, lung squamous cell carcinoma; KSCC, keratinizing squamous cell carcinoma; NKSCC, non-keratinizing squamous cell carcinoma; BSCC, basaloid squamous cell carcinoma.

We conducted a multivariable exclusivity and co-occurrence analysis for LUSC, KSCC, and NKSCC respectively. Cases with BSCC were excluded due to limited sample size. Results showed that PIK3CA alterations strongly correlated with aberrations of PTCH1/FGFR1/CDKN2A; KRAS and FGFR1/CDKN2A/JAK1/KIT co-alterations were frequently observed in LUSC (**Figure 4A**). Of note, cases with TP53 alterations had less EGFR alterations in KSCC (P = 0.013, OR = 0.158), while this difference was not found in NKSCC (**Figure 4B**). In NKSCC, we detected CCND1/FGF3/FGF4/FGF19 and RET/JAK1/PTEN/FGFR1 co-alterations (**Figure 4C**).

All genes and samples were clustered into four subgroups using NMF clustering method based on the somatic mutations and CNAs. Cluster 1 revealed a higher mutation frequency of PIK3CA and CDKN2A compared with the other clusters, while Cluster 2 was enriched in CCND1/FGF19/FGF3/FGF4 amplifications. As for Cluster 3, FGFR1 amplifications and EGFR and BRCA2 mutations were more frequent. Almost all the cases in Cluster 4 harbored TP53 mutations and less gene amplifications. Interestingly, we found BSCC congregated in this subgroup (**Figure 4D**).

### Comparative Mutational Analysis of LUSC in Our Cohort and TCGA Cohort

**Figure 5:**
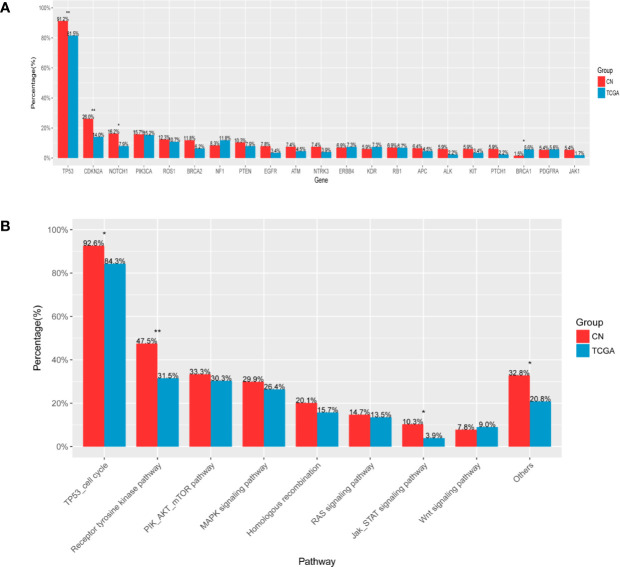
Comparative mutational analysis of LUSC in our cohort and TCGA cohort **(A)**. X-axis denotes gene, Y-axis, patient frequency **(B)**. X-axis denotes signaling pathway, Y-axis, patient frequency. *p < 0.05, **p < 0.01.

The dataset from TCGA comprised of 504 LUSC samples, which were generated through whole exome sequencing, accordingly, comparative analyses of LUSC in our cohort and data obtained from the completely nonoverlapping TCGA cohort were only limited to single nucleotide variants and excluded copy number alterations and gene rearrangements. In our cohort, mutation frequencies of TP53, CDKN2A, and NOTCH1 were higher (p = 0.006, p = 0.005, p = 0.018, respectively), while BRCA1 were lower (p = 0.043) (**Figure 5A**). For signaling pathway, mutations of TP53 cell cycle, Receptor tyrosine kinase pathway and JAK-STAT signaling pathway were more frequent in our cohort (p = 0.014, p = 0.002, p = 0.018, respectively) (**Figure 5B**).

### Molecular Prognosis Factors Among LUSC Subtypes

**Figure 6:**
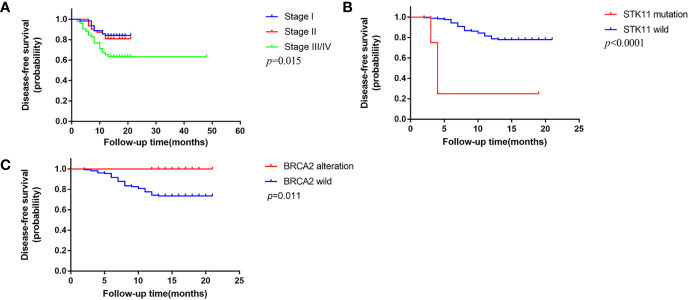
One-year disease-free survival (DFS) in this cohort **(A)**. DFS based on pathologic stage **(B)**. DFS based on STK11 status **(C)**. DFS based on BRCA2 status.

We followed up all patients every three months consecutively for 1 year, including 6 patients died of other causes, 20 patients failed to follow up. The date of last follow-up was January 31, 2020 and the median follow up was 14 months. A total of 178 patients were included for further prognosis analysis. Forty-one (23.0%) patients suffered a relapse in 1 year, among which 21 (21.4%) were KSCC, 16 (22.9%) were NKSCC, and 4 (40%) were BSCC. We employed bivariate Cox proportional hazards models to explore the clinical and genetic prognosis factors among LUSC subtypes. Univariable analysis revealed that pathological stage (p = 0.011), STK11 alterations (p < 0.001), and TOP2A alterations (p = 0.001) were in connection with a risk of recurrence in the patients with LUSC. IDH1 alterations (p = 0.028) and TOP2A alterations (p = 0.003) were in connection with a higher risk of recurrence in the patients with KSCC; higher pathological stage (p = 0.01), BRCA2 alterations (p = 0.098) and TOP2A alterations (p = 0.073) were in connection with a higher risk of recurrence in the patients with NKSCC. Further multivariable analysis showed that higher pathological stage (HR, 1.635; p = 0.015), STK11 alterations (HR, 13.266; p < 0.001), and TOP2A alterations (HR, 4.759; p = 0.004) were relevant to higher risk of recurrence in the patients with LUSC. TOP2A alterations were relevant to higher risk of recurrence in the patients with KSCC (HR, 6.490; p = 0.003), while no statistic differences of correlation occurred in the patients with NKSCC. The 1-year DFS of stage I, stage II, and stage III/IV subgroups were 84.0, 80.9, and 63.4% respectively (P = 0.015, **Figure 6A**). The 1-year DFS of STK11 alterations and STK11 wild subgroups were 25.0 and 78.0% (p < 0.001, **Figure 6B**). The 1-year DFS of BRCA2 alterations and BRCA2 wild subgroups were 0 and 26.1% (p = 0.011, **Figure 6C**). No difference in 1-year DFS among three subtypes was observed in our study.

## Discussion

The last decade has witnessed a remarkable advance to molecular-based classification and precise medicine, in which combination of genomic alterations and histopathology has been applied to clinical practice for better management of lung cancer. Given that few systematic comparison of genetic profiles has yet been reported for different subtypes in Chinese patients with LUSC, we conducted this study to elucidate the molecular characteristics of different subtypes of LUSC. As far as we know, this is the largest study illuminating genetic spectra of LUSC in more than 200 Chinese individuals based on pathological subtypes.

The overwhelming majority of the patients were males (92.2%), former or current smokers (80.4%), and older people (75.5%), which was generally in accordance with acknowledged epidemiology of LUSC. Tobacco exposure is of close association with LUSC, increasing burden of somatic mutations (16). In line with previous studies, our results demonstrated that majority of patients with LUSC were former or current smokers, especially in KSCC (17). With regard to genomic profiling, nearly all patients with LUSC harbored genetic alterations including somatic mutations or CNAs, prompting high mutation burden in LUSC. We found that FGFR alterations were more common in patients diagnosed with stage II, compared with other stages. Limited by sample sizes and the low frequency of alterations, we cannot put forward any conclusion.

In this study, we found generally congruent mutation frequencies of different subtypes in TOP 20 genes including TP53 (93.9% in KSCC, 88.5% in NKSCC, 100% in BSCC), CDKN2A (28.1% in KSCC, 25.6% in NKSCC, 30.8% in BSCC), and NOTCH1 (15.8% in KSCC, 15.4% in NKSCC, 30.8% in BSCC), however, BSCC represented relatively lower frequency of PIK3CA mutations compared to the other two subtypes (16.7% in KSCC, 16.7% in NKSCC, 0% in BSCC). Kim et al. (9) reported that seven genes displayed statistical enrichment for mutation: TP53, RB1, PTEN, NFE2L2, KEAP1, MLL2, and PIK3CA. Similarly, Zhang et al (18). found the most frequently mutated gene was TP53 (81.1%) in LUSC, which was in accordance with our study. Interestingly, another study including 157 Chinese patients with resected LUSC reported mutation frequencies of 56.1% for TP53, 8.9% for CDKN2A, 8.9% for PIK3CA (7), and the incidence were significantly lower than those observed in our study. As reported by a recent study, TP53 mutations were detected in almost all LUSC patients (90%) and CDKN2A mutations accounted for 19.4% of LUSC patients. Meanwhile, TP53 mutants only occurred in 81% of NKSCC patients, which is generally consistent with our study (19). It was reported that progression-free survival (PFS) and overall survival (OS) in immunotherapy-treated patients harboring TP53 mutations/STK11-EGFR-wild type tumors prolonged (20). It could probably explain why LUSC patients have strong adaptive immune response to immunotherapy. However, this research only included LUAD patients. Additional studies including more LUSC patients are needed to investigate robust molecular biomarkers identifying best responders to immunotherapy. Another study including TCGA cohorts proposed that losses of CDKN2A increased the susceptibility to resistance of IFNγ and immunotherapy by concomitant losses of JAK2 in LUSC. It observed that the majority of samples harboring losses in JAK2 showed concurrent CDKN2A and JAK2 losses in 90.5% of LUSC (21), which was inconsistent with our study. Ethnic diversity and different detection methods may lead to the contradiction.

We further explored somatic mutations and CNAs in different subtypes, respectively. We found that FGFR1, SMO and ARAF somatic mutations showed statistic differences among LUSC pathologic subtypes, however, differences did not exist between any two subtypes. As for CNAs, our findings demonstrated that FGFR1 amplifications occurred more frequently in NKSCC than KSCC with statistic difference and compared with NKSCC, IGF1R amplifications were more frequent in BSCC. Heist et al. (22) identified that FGFR1 amplifications were found in 16% of LUSC patients and FGFR1 amplifications status had no correlation with age, sex, staging, histologic subtype, smoking history. YEO et al. (23) reported that IGF-1R was closely associated with smoking status and highly expressed in LUSC compared to other types of NSCLC, furthermore, elevated expression of IGF-1R was significantly related to poor clinical outcome.

We also explored the differences of signaling pathway mutations among different LUSC subtypes. Incidence of RAS signaling pathway mutations was more frequent in NKSCC compared with KSCC. Ras signaling pathway are crucial to the initiation or progression of the cancer. Numerous studies demonstrated that indole derivatives can target Ras proteins or Ras-related proteins to block the transmission of the Ras-Related signaling pathway and have broad prospect for therapy (24).

Exclusivity and co-occurrence of mutations revealed that cases with TP53 alterations had less EGFR alterations in KSCC (P= 0.013, OR= 0.158), while not in NKSCC. As reported, in LUSC patients, co-occurrence of mutations including STK11/MTOR (OR > 10, P < 0.001) and TP53/CDKN2A (OR > 10, P < 0.001) were identified. Furthermore, no exclusivity of mutations was identified (18).The contradictory results may be attributed to different OncoScreen panels and analytical methods. NMF clustering of all genes based on the somatic mutations and CNAs exhibited that BSCC may have unique genetic spectra. Due to limited sample sizes, different detection methods and classification standards, we cannot safely draw a conclusion.

Compared with TCGA cohorts, Chinese cohorts exhibited some statistic differences in both somatic mutations and signaling pathway, which further verified that ethnic diversity had some connection with the pathogenesis of lung cancer (25). We found mutation frequencies of TP53, CDKN2A, and NOTCH1 were higher than TCGA cohorts which were congruent with a previous study (18). Contradictorily, Izumi M et al. (26) recently reported that among LUSC patients, TP53 and PIK3CA mutations were more common in TCGA cohorts. Another study comparing the Korean cohorts with TCGA cohorts found that both cohorts displayed a similarly high frequency of mutations of TP53 and NOTCH1, but CDKN2A mutations were observed more frequently in TCGA cohorts (9). It could prompt that even in Asian subjects, there still exist some genetic differences in different countries. Further extensive researches will assist in better elucidating genomic differences between sub-ethnic groups.

Multivariable analysis displayed that higher pathological stage, STK11 alterations and TOP2A alterations were relevant to a higher risk of recurrence in the patients with LUSC. Further analysis demonstrated that TOP2A alterations were related to a higher risk of recurrence in the patients with KSCC, neither in NKSCC nor in BSCC. As for pathological stage, Chen et al. (27) also reported the similar finding previously. A recent study identified TOP2A as a negative prognostic factor in LUAD (28), but the prognostic value of TOP2A in LUSC remains obscure. STK11 is a frequently mutated gene and has been identified as an important suppressor in LUSC (29). A recent study reported a trend toward a reduced PFS in LUAD patients harboring STK11 mutations (20). Worse OS and PFS outcomes were also observed in NSCLC patients with STK11 mutations receiving immunotherapy or chemotherapy (30). Interestingly, Bange et al. (31) found co-mutations of STK11 with TP53 was associated with a better prognosis. As a rare subtype, prognosis of BSCC remains obscure. We did not observe statistic differences in 1-year DFS among three subtypes. Previous studies demonstrated patients with BSCC had a poor prognosis compared to the other two subtypes (32, 33). Amplifications of genes at 11q13 involving CCND1, FGF3, FGF4, and FGF19 displayed differences between BSCC and other two subtypes. A study reported patients with gains of 11q13.1 had a poor survival (34). However, a recent propensity score matching (PSM) analysis reported that patients with BSCC had a better prognosis than those with KSCC or NKSCC (35). Further analysis of long-term survival time based on larger cohorts is in urgent need to determine whether prognosis of BSCC truly differs from the other two subtypes.

There still remained some limitations in our study. First of all, insufficient sample size limited the ability to perform comparative analyses of less common genomic alterations and prognosis. Secondly, only the resectable cases of LUSC were involved, leading to skew the associations of pathologic staging and genetic alterations. In view of the complexities of the genomic landscape, larger scale multi‐center studies are required to clarify the mutation profiles and subtle genetic differences of LUSC based on histopathologic subtypes. Thirdly, different post-surgery treatments could influence the prognostic impact of genetic alterations. Further prospective studies are in great need to analyze prognostic effect of genomic alterations which take treatments into account. Finally, due to the short follow-up period, we only analyzed 1-year DFS of these patients. We will continue to follow up for further analysis.

In summary, our study comparatively described the genomic characteristics of surgically resected LUSC subtypes in a relatively large cohort of Chinese patients. It revealed the commonality in LUSC subtypes and identified several differences among them meanwhile, which could assist management of LUSC patients.

## Data Availability Statement

The datasets presented in this study can be found in online repositories. The names of the repository/repositories and accession number(s) can be found below: NODE, accession: OEP001258 (http://www.biosino.org/node/project/detail/OEP001258).

## Ethics Statement

The studies involving human participants were reviewed and approved by the institutional review board of Shanghai Chest Hospital. The patients/participants provided their written informed consent to participate in this study.

## Author Contributions

JQ collected the data, provided statistical analysis, and wrote the original draft. RC assisted in data analysis. RZ assisted in collecting the data. YH and YY conceived and designed the study. All authors contributed to the article and approved the submitted version.

## Funding

This work was supported by grants from Shanghai Municipal Science and Technology Commission Research Project (16431903200).

## Conflict of Interest

The authors declare that the research was conducted in the absence of any commercial or financial relationships that could be construed as a potential conflict of interest.
