# Supplementary material for: Comprehensive Molecular Characterizations of Chinese Patients With Different Subtypes of Lung Squamous Cell Carcinoma
Source: Front Oncol. 2020 Dec 10;10:607130. doi: 10.3389/fonc.2020.607130 (PMC7758445; doi:10.3389/fonc.2020.607130)
Supplement: Supplementary file 1 [file DataSheet_1.pdf]

Supplementary Material

Supplemental Table 1. OncoScreen 68 genes list

ALK	BRAF	EGFR	ERBB2
KRAS	MET	RET	ROS1
AKT1	APC	AR	ARAF
ATM	AXL	BCL2L11	BRCA1
BRCA2	CCND1	CD74	CDK4
CDK6	CDKN2A	CTNNB1	DDR2
ERBB3	ESR1	ERBB4	FGF19
FGF3	FGF4	FGFR1	FGFR2
FGFR3	FLT3	HRAS	IDH1
IDH2	IFG1R	JAK1	JAK2
KDR	KIT	MAP2K1	MTOR
MYC	NF1	NOTCH1	NRAS
NRG1	NTRK1	NTRK2	NTRK3
PDGFRA	PIK3CA	PTCH1	PTEN
RAF1	RB1	SMAD4	SMO
STK11	TOP2A	TP53	TSC1
TSC2	CYP2D6	DPYD	UGT1A1

Supplemental Table 2. Differences of genetic profiles among LUSC subtypes

	KSCC	NKSCC	BSCC	P value
FGFR1	0.0%	3.8%	7.7%	0.035
ARAF	0.0%	2.6%	7.7%	0.038
SMO	0.0%	2.6%	7.7%	0.038
FGFR1 Amplification	11.4%	26.9%	7.7%	0.015
IGF1R Amplification	17.5%	0.0%	15.4%	0.011

Abbreviations: LUSC, lung squamous cell carcinoma; KSCC, keratinizing squamous cell carcinoma; NKSCC, nonkeratinizing squamous cell carcinoma; BSCC, basaloid squamous cell carcinoma

Supplemental Table 3. Differences of genetic profiles between KSCC and NKSCC

	KSCC	NKSCC	P value
FGFR1	0.0%	3.8%	0.066
ARAF	0.0%	2.6%	0.164
SMO	0.0%	2.6%	0.164
FGFR1 Amplification	11.4%	26.9%	0.007
IGF1R Amplification	1.8%	0.0%	0.515

Abbreviations: KSCC, keratinizing squamous cell carcinoma; NKSCC, nonkeratinizing squamous cell carcinoma

Supplemental Table 4. Differences of genetic profiles between KSCC and BSCC

	KSCC	BSCC	P value
FGFR1	0.0%	7.7%	0.102
ARAF	0.0%	7.7%	0.102
SMO	0.0%	7.7%	0.102
FGFR1 Amplification	11.4%	7.7%	1.000
IGF1R Amplification	1.8%	15.4%	0.052

Abbreviations: KSCC, keratinizing squamous cell carcinoma; BSCC, basaloid squamous cell carcinoma

Supplemental Table 5. Differences of genetic profiles between NKSCC and BSCC

	NKSCC	BSCC	P value
FGFR1	3.8%	7.7%	0.466
ARAF	2.6%	7.7%	0.374
SMO	2.6%	7.7%	0.374
FGFR1 Amplification	26.9%	7.7%	0.176
IGF1R Amplification	0.0%	15.4%	0.019

Abbreviations: NKSCC, nonkeratinizing squamous cell carcinoma; BSCC, basaloid squamous cell carcinoma

Supplemental Table 6. Differences of genetic profiles based on stage

Stage	FGFR1 wt	FGFR1 mut	P value
I	100%	0%	
II	94.6%	5.4%	0.038
III/IV	100%	0%	

Abbreviations: wt, wild type; mut,mutant type

Supplemental Table 7. Differences of genetic profiles based on gender

	Male	Female	P value
TP53	93.6%	68.8%	0.005
SMAD4	0.5%	12.5%	0.017
FLT3	3.7%	18.8%	0.034
ARAF	0.5%	12.5%	0.017

Supplemental Table 8. Differences of genetic profiles based on smoking status

Smoking status	TP53 wt	TP53 mut	P value
Nonsmoker	22.5%	77.5%	0.001
Smoker	4.9%	95.1%	

Abbreviations: wt, wild type; mut, mutant type

Supplemental Table 9. Differences of genetic profiles based on age

Age	AXL wt	AXL mut	P value
≥60	99.4%	0.6%	0.046
< 60	94.0%	6.0%	

Abbreviations: wt, wild type; mut, mutant type

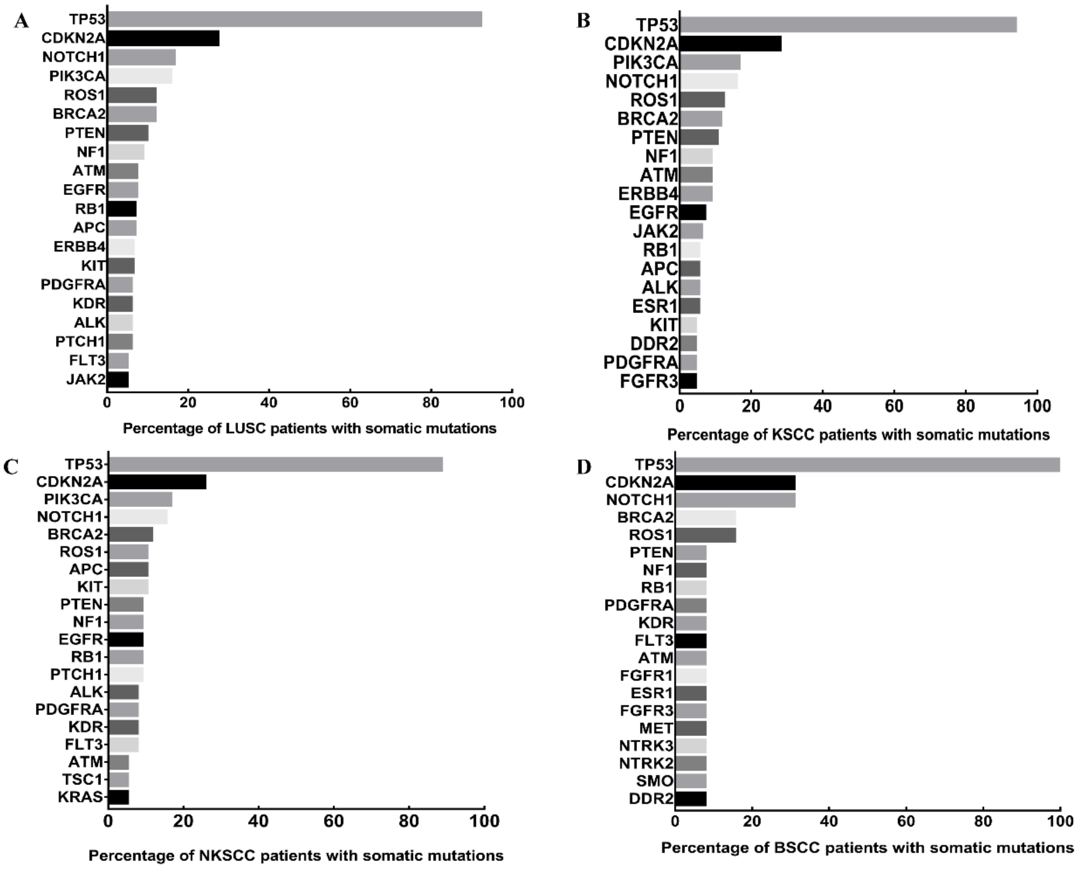

Supplemental Figure 1. Prevalence of TOP20 genes in Chinese patients with (A) LUSC, (B) KSCC, (C) NKSCC, (D) BSCC. X-axis denotes patient frequency, Y-axis, TOP20 genes. LUSC, lung squamous cell carcinoma; KSCC, keratinizing squamous cell carcinoma; NKSCC, nonkeratinizing squamous cell carcinoma; BSCC, basaloid squamous cell carcinoma.

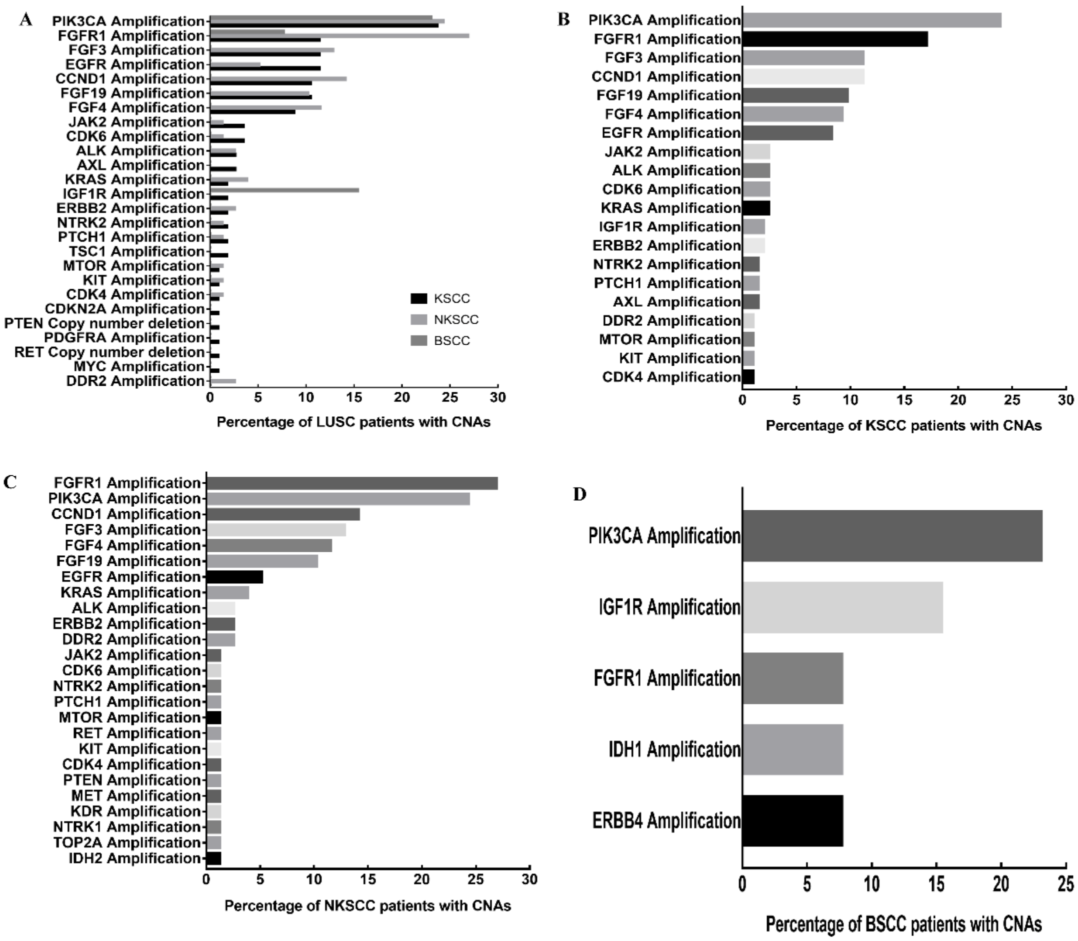

Supplemental Figure 2. Prevalence of the most commonly detected CNAs in Chinese patients with (A) LUSC, (B) KSCC, (C) NKSCC, (D) BSCC. X-axis denotes patient frequency, Y-axis, TOP20 genes. LUSC, lung squamous cell carcinoma; KSCC, keratinizing squamous cell carcinoma; NKSCC, nonkeratinizing squamous cell carcinoma; BSCC, basaloid squamous cell carcinoma.

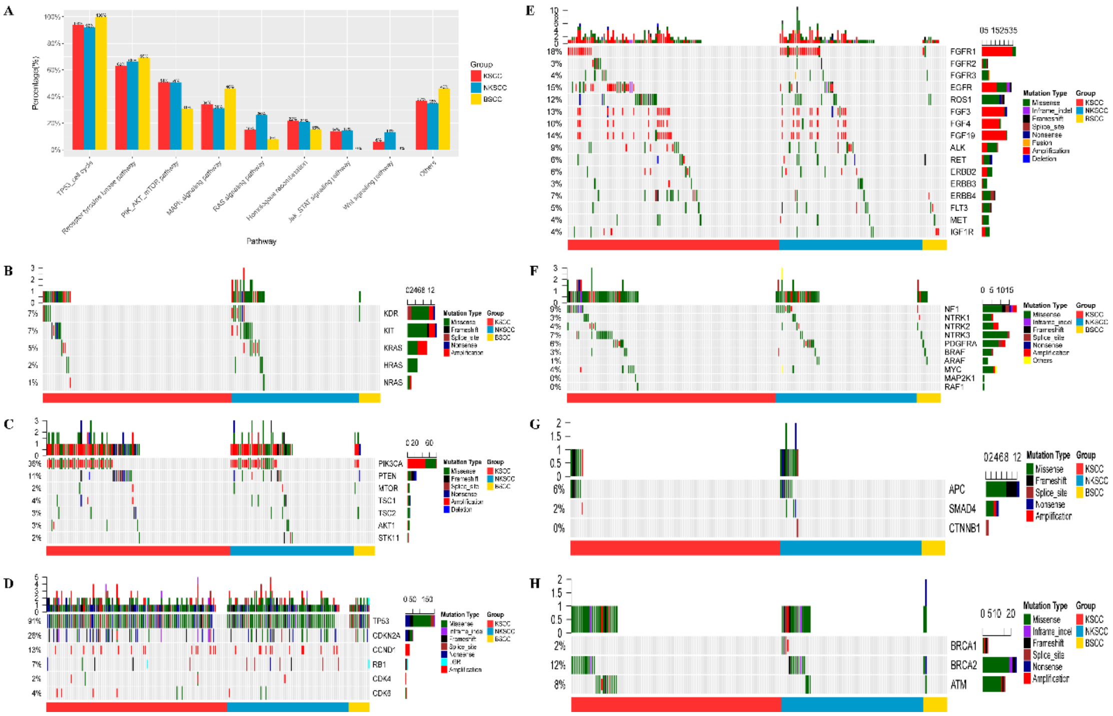

Supplemental Figure 3. (A) Prevalence of signaling pathway mutations including (B) RAS signaling pathway, (C) PIK-AKT-mTOR pathway, (D) TP53-cell cycle, (E) Receptor tyrosine kinase pathway, (F) MAPK signaling pathway, (G) Wnt signaling pathway and (H) Homologous recombination in different subtypes of LUSC. Each column represents a patient and each row represents a gene. Column on the left represents the mutation rate of each gene. Column on the right represents mutated genes. Top plot represents the overall number of mutations a patient carried. Different colors denote different types of mutation. KSCC, keratinizing squamous cell carcinoma; NKSCC, nonkeratinizing squamous cell carcinoma; BSCC, basaloid squamous cell carcinoma. KSCC, keratinizing squamous cell carcinoma; NKSCC, nonkeratinizing squamous cell carcinoma; BSCC, basaloid squamous cell carcinoma
